# Standard versus extralevator abdominoperineal excision and oncologic outcomes for patients with distal rectal cancer: A meta-analysis

**DOI:** 10.1097/MD.0000000000009150

**Published:** 2017-12-29

**Authors:** Yunfeng Zhang, Duo Wang, Lizhe Zhu, Bin Wang, Xiaoxia Ma, Bohui Shi, Yu Yan, Can Zhou

**Affiliations:** a Department of the Second Thoracic Surgery, the First Affiliated Hospital of Xi’an Jiaotong University; b Department of General Surgery, the Second Affiliated Hospital of Xi’an Medical College; c Department of Breast Surgery, the First Affiliated Hospital of Xi’an Jiaotong University, Xi’an, Shaanxi Province, China.

**Keywords:** circumferential resection margin, extralevator abdominoperineal excision, intraoperative bowel perforation, local recurrence, rectal cancer

## Abstract

**Background:**

The role of extralevator abdominoperineal excision (ELAPE) for distal rectal cancer remains controversial, and the procedure is not widely accepted or practiced.

**Methods:**

An electronic search of Medline, EMBASE, Web of Science, and similar databases for articles in English was performed from the inception of the study until October 31, 2017. Two reviewers extracted information and independently assessed the quality of included studies by the methodological index for nonrandomized studies, then data were analyzed with Review Manager 5.3 software and Stata version 12.0 software.

**Results:**

Our meta-analysis included 17 studies with 3479 patients, of whom 1915 (55.0%) underwent ELAPE and 1564 (44.0%) underwent abdominoperineal excision (APE). Compared with patients undergoing APE, patients undergoing ELAPE had a significant reduced risk of no more than 3 years local recurrence (LR) (risk ratio [RR] = 0.27, 95% confidence interval [CI] = 0.08–0.94), 3-year mortality (odds ratio [OR] = 0.45, 95% CI = 0.20–0.97), intraoperative bowel perforation (IBP) involvement (RR = 0.48, 95% CI = 0.31–0.74), and circumferential resection margin (CRM) positivity (RR = 0.66, 95% CI = 0.43–1.00) at the threshold level.

**Conclusions:**

The application of ELAPE is more effective in reducing the chance of 3 years LR, mortality, IBP involvement and CRM positivity than conventional APE, and worthy of being widely applied in surgical treatment of the distal rectal cancer.

## Abstract 2

Supplemental Digital Content is available in the text

## 1 Introduction

Despite combination with adjuvant therapy, radiotherapy, and chemotherapy, the distal rectal cancer can be mostly treated with surgery. Abdominoperineal excision (APE) is a surgical treatment for patients with distal rectal cancer in whom an anterior resection cannot be performed. It has been the standard operation for advanced distal rectal cancer. However, a high rate of intraoperative bowel perforation (IBP) as well as circumferential resection margin (CRM), strong predictors of survival in rectal cancer patients,^[1]^ has also been consistently reported.^[2–6]^ Therefore, the APE is mainly performed in patients whose tumors situated close to the dentate line. The underlying reason may be that the resected specimens usually narrow at the lower border of the mesorectum and at the level just above the levator muscle when performing a conventional APE. Hypothetically, a wider excision would reduce these events and, hence, the risk of local recurrence (LR). Surgeons tried to remove the increased tissue in the distal rectum and en bloc excision of the levator ani.^[7]^

With the better understanding of the disease spread in the past several decades, there are significant advances in the surgical techniques of distal rectal cancer. Extralevator abdominoperineal excision (ELAPE, also known as cylindrical APE, CAPE), first reported by West in 2008^[7–16]^ and aroused the concern of colorectal surgeon. This increased interest was attributed to its superiority in terms of reduced risk of CRM positivity,^[11]^ IBP involvement and LR compared with conventional APE.^[11,17]^ Nevertheless, an increasing number of reports have shown that the application of ELAPE produced inconclusive conclusions of the long-term survival, such as mortality and rate of LR, and resulted in higher morbidity and postoperative complications including infection and perineal hernia,^[8,12–16,18–25]^ due to insufficient sample size. Thus, the relevance of ELAPE in terms of LR, mortality or CRM positivity has not been proven. For this reason, we conducted a systematic review of the literature and meta-analysis with a sufficient sample size (n = 3479) to comprehensively assess the efficacy of ELAPE based on CRM, IBP, LR, and long-term survival rate.

## 2 Methods

This review protocol was registered and published in the International Prospective Register of Systematic Reviews, PROSPERO (CRD42013006206), and followed the prescribed steps therein.^[26]^ This report complies with the preferred reporting items for systematic reviews and meta-analyses (PRISMA). All randomized and nonrandomized case–control studies that followed the study selection below were included into this meta-analysis. Furthermore, we appraised the studies’ data quality assessment using the methodological index for nonrandomized studies (MINORS). Due to this study was a meta-analysis of 17 studies, ethical approval was not necessary

### 2.1 Data sources and searches

First, an electronic search of MEDLINE, EMBASE, Wiley Online Library and the Cochrane Library, was performed from the inception of the study to October 31, 2017, using the terms “extralevator/cylindrical/extended abdominoperineal excision/resection,” ELAPE, CAPE, and “rectal cancer.”

### 2.2 Study selection

The included studies had to be published in English and meet the following criteria: randomized or nonrandomized controlled study with parallel controls; comparisons of LR, mortality or CRM, IBP; laparoscopic or hand-assisted resections; and grey literatures, such as conference proceedings, reports, and other peer-reviewed research.

Publications with the following characteristics were excluded: the outcomes of interest were not reported, or it was impossible to calculate the outcomes from the published results; the study did not include a distinct group of patients or comparisons of the outcomes of interest; and review articles.

### 2.3 Outcomes definition

ELAPE was abbreviated to ELAPE. APE was defined as APE. LR was abbreviated as LR. Intraoperative bowel perforation was abbreviated as IBP. CRM was abbreviated as CRM.

### 2.4 Data collection and quality assessment

Data were extracted from the original studies by 2 independent reviewers who were blinded to journal names, institutions, and funding grants using a standardized form. Disagreements regarding inclusion were discussed with the guidance of the corresponding author via e-mail, if necessary. If no response was received, a second e-mail was sent 1 week later.

To ascertain the validity of the eligible studies, the quality of each report was appraised based on the 12 items described in the methodological index for MINORS. The total quality scores ranged from 0 (low quality) to 24 (high quality). Disagreements were resolved by discussion with the corresponding author via e-mail or personal interview.

### 2.5 Data synthesis and analysis

The primary outcome was the long-term survival, such as mortality and rate of LR, as they were the most frequently reported parameters of clinical utility in colorectal surgery. Secondary outcomes included CRM positivity, IBP involvement, because they are important indices of perioperative recovery. Other outcomes of interest, such as and genitourinary system complications, were not analyzed because they were included in a minority of published studies or participants.

For each outcome of interest, the effect sizes of the individual studies were pooled using fixed or random-effects models with Review Manager 5.3 software.^[27,28]^ Heterogeneity was examined by computing the I-squared statistic.^[29,30]^ If the heterogeneity was high^[30]^ (*I*^2^ > 50% or *P* < .10), sensitivity analysis, performed with Stata version 12.0 software (Stata Corp LP, College Station, TX), and subgroup analysis were performed to find out potential origin of heterogeneity.

Funnel plot were used for diagnosis of potential publication bias,^[31]^ performed with Review Manager 5.3 software or Stata version 12.0 software (Stata Corp LP). In addition, the possible effect of publication bias in our meta-analysis was further assessed using Duval and Tweedie nonparametric “trim and fill” procedure.^[32]^

## 3 Results

### 3.1 Selected studies and methodological quality

**Figure 1:**
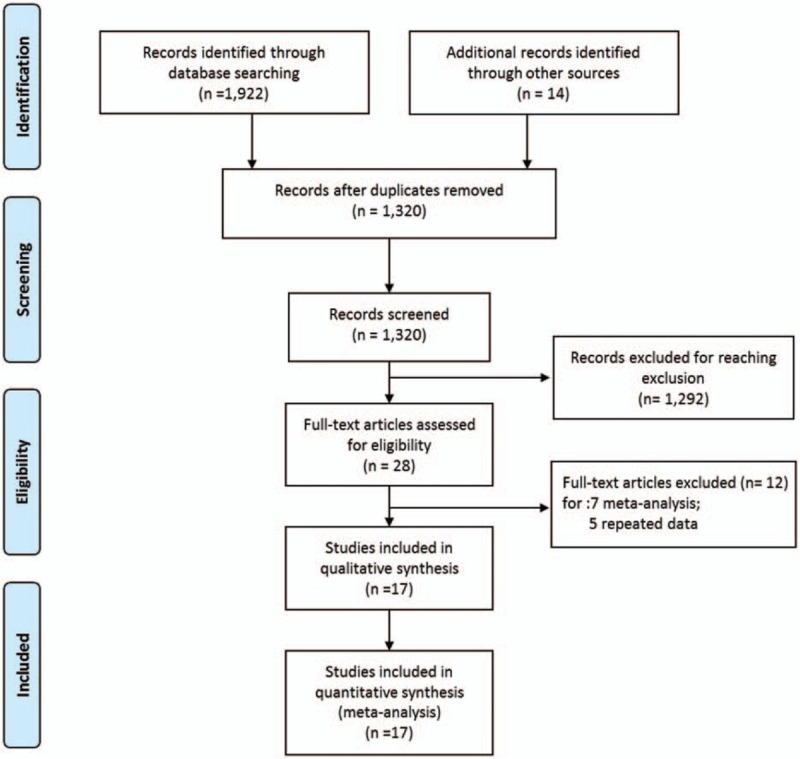
Flow diagram of the search and selection method.

Figure 1 shows a flow diagram of our search and selection process. Seventeen^[11–14,16,17,19–21,33–40]^ of 28 studies^[6,11–17,19–21,23–25,33–46]^ were selected: 7 studies were excluded because they were meta-analyses or systematic reviews^[23–25,39,41–44]^; the results of the study by West et al were reported in 2 papers,^[6,11]^ and thus, the study published by the *Journal of Clinical Oncology* in 2008 was excluded^[6]^; the results reported by Asplund,^[15]^ Prytz^[20,45]^ and Angenete^[37]^ were from the same institution, and the study by Asplund^[15]^ was excluded because is overlapping in the time period with the other 2 studies. In addition, the study by Zhang^[46]^ was excluded for being from the same research institute and the same database as the study by Shen.^[35]^ The results of the methodological quality evaluation are shown in Table 1. The total quality scores of the included studies ranged from 14 to 20 scores (Table 1). None of the included studies performed a prospective calculation of the study size or an unbiased assessment of the study outcomes. A randomized controlled design was performed in only one study.^[14]^

**Table 1:**
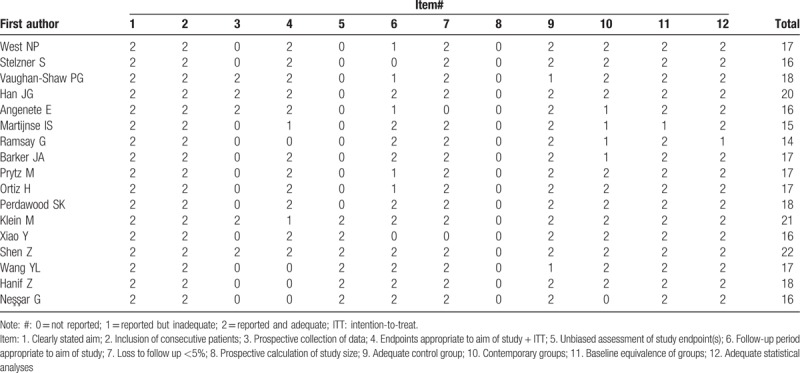
Methodological quality assessment of the included studies.

### 3.2 Characteristics of the studies and patients

The selected trials included a total of 17 studies and 3479 patients, of whom 1915 (55.0%) underwent ELAPE, and 1564 (44.0%) underwent APE (Table 2). Among the 17 studies, only 1 was a randomized controlled trial (RCT),^[14]^ 5 studies were performed in the United Kingdom,^[11,13,16,21,40]^ 4 in China,^[14,31–32,35–36,47]^ 2 in Sweden,^[20,37]^ 2 in Denmark,^[33–34]^ and the remaining in Germany,^[11–14,16,17,19–21,33–40]^ Netherlands,^[11–14,16–17,19–21,33–40]^ Spain,^[11–14,16–17,19–21,33–40]^ and Turkey.^[11–14,16–17,19–21,33–40]^

**Table 2:**
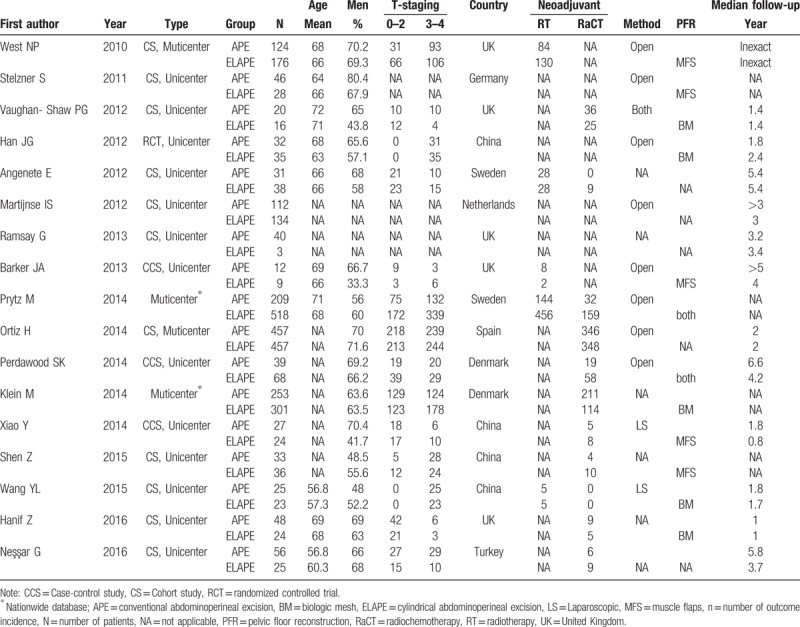
Characteristics and demographics of the included studies.

In addition, 13 studies investigated CRM as an outcome measure, 11 studies investigated IBP, 9 investigated LR, 4 studies investigated long-term survival (Table 3). A large variation in the different studies in terms of follow-up duration was observed, ranging from 0.8 year to more than 5 year. Large variations were also observed in T staging of the tumor; the majority of the studies reported T0–T4 tumors (Table 2).

**Table 3:**
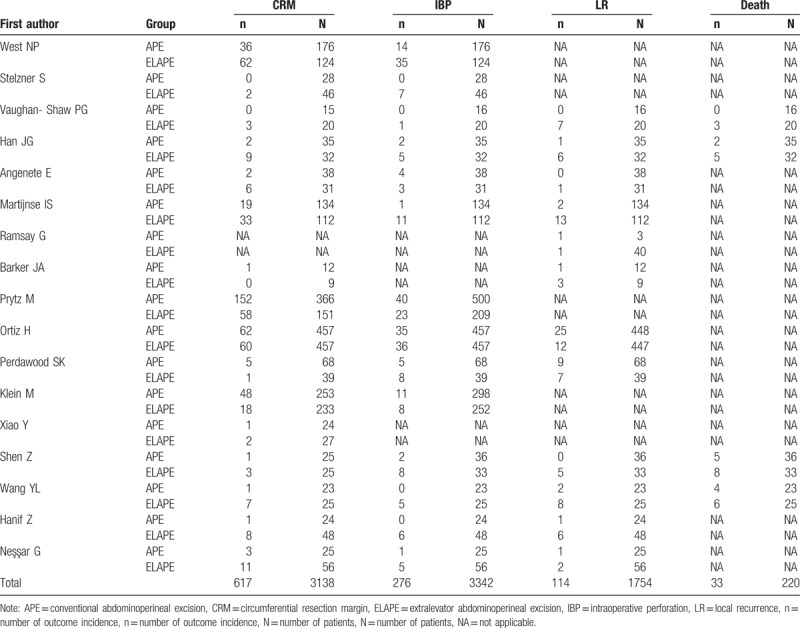
Outcomes of interest in the included studies.

### 3.3 Long-term clinical efficacy of ELAPE versus APE

**Figure 2:**
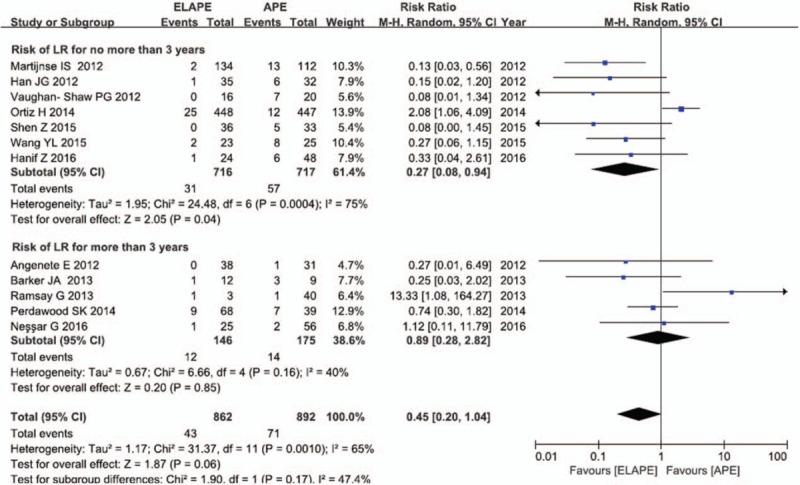
Risk of local recurrence between ELAPE and APE. APE = abdominoperineal excision, ELAPE = extralevator abdominoperineal excision.

Data describing the effect of ELAPE on LR were available for 12 studies with 1754 participants, with the overall LR rate of 6.50% (114/1754). A pooled risk ratio (RR) of 0.45 (95% confidence interval [CI] = 0.20–1.04), using a random effects model (Fig. 2), due to high heterogeneity (*I*^2^ = 65%, *P* < .10), demonstrated that the ELAPE procedure had a tendency to reduce the risk of LR when compared with APE. After subgroup analysis, the pooled RR of 0.27 (95% CI = 0.08–0.94) that ELAPE procedure may reduce increase the risk of LR for no more than 3 years, also with high heterogeneity (*I*^2^ = 75%, *P* < .10).

**Figure 3:**
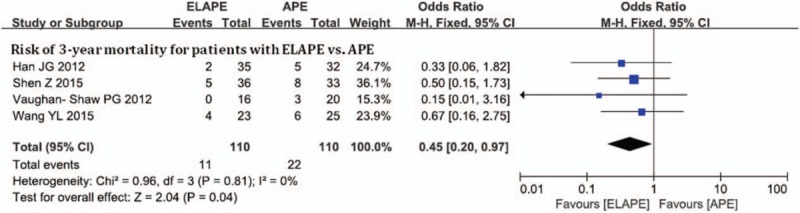
Risk of 3-year mortality between ELAPE and APE. APE = abdominoperineal excision, ELAPE = extralevator abdominoperineal excision.

Four studies with 220 participants investigated survive as an outcome measure, with the 3-year mortality of 15% (33/220). As shown in Figure 3, the pooled OR of 0.45 (95% CI = 0.20–0.97) revealed a significant reduction in 3-year mortality for rectal cancer patients with no heterogeneity (*I*^2^ = 0%, *P* = .81).

### 3.4 Short-term clinical efficacy of ELAPE versus APE

**Figure 4:**
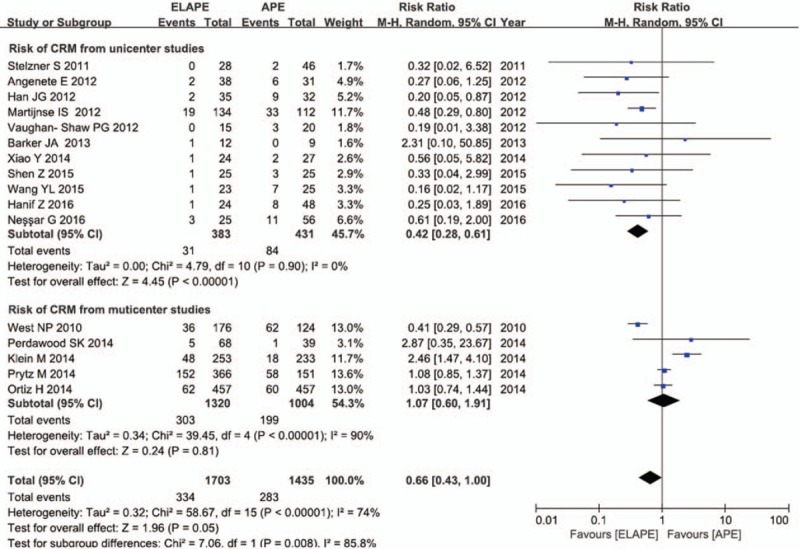
Risk of CRM between ELAPE and APE. APE = abdominoperineal excision, CRM = circumferential resection margin, ELAPE = extralevator abdominoperineal excision,

Sixteen studies with 3138 participants investigated CRM as an outcome measure, with the overall CRM rate of 19.7% (617/3,138). The pooled RR of 0.66 (95% CI = 0.43–1.00) in our meta-analysis using a random effects model (Fig. 4), due to high heterogeneity (*I*^2^ = 85.8%, *P* = .008 < 0.10), suggested an insignificant difference in the risk of CRM by a third at the threshold level. After subgroup analysis, we found that multicenter studies were the main causes of the heterogeneity. A significant difference in the reduced risk of CRM by ELAPE was observed after excluding studies by multicenter studies (RR = 0.42, 95% CI = 0.28–0.61), with no heterogeneity (*I*^2^ = 0%, *P* = .90).

**Figure 5:**
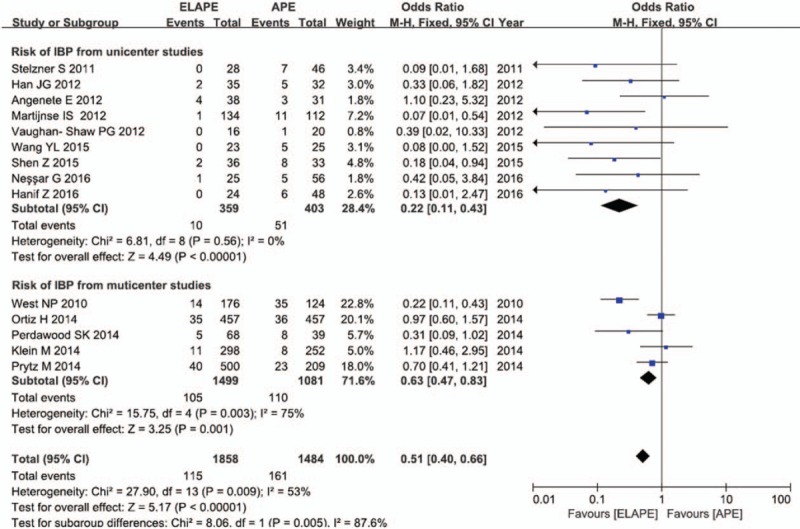
Risk of IBP between ELAPE and APE. APE = abdominoperineal excision, ELAPE = extralevator abdominoperineal excision, IBP = intraoperative bowel perforation.

Data for the effect of ELAPE on IBP were available for 14 studies with 3342 participants, with the overall IBP rate of 18.5% (617/3342). As shown in Figure 5, the pooled RR of 0.48 (95% CI = 0.31–0.74) for IBP comparing ELAPE with APE, indicated that the application of IBP could reduce the risk of IBP more than 50 percent, with high heterogeneity (*I*^2^ = 63.0%, *P* = .10). A consistent result (RR = 0.30, 95% CI = 0.16–0.59), with no significant heterogeneity (*I*^2^ = 0%, *P* = .45 > .05), obtained after excluding the multicenter studies, demonstrated that ELAPE intervention was associated with a 50% lower risk IBP.

### 3.5 Publication bias analysis

Funnel plots were used to assess the publication bias of the included studies. The asymmetries shown in the funnel plots figures S1a, S1c, S1d and S1e revealed the possibility of publication bias. Because of this, we undertook a sensitivity analysis using the trim and fill method by Stata version 12.0 software, with the aim to impute hypothetical negative unpublished studies to mirror the positive studies that cause funnel plot asymmetry.^[32]^ The pooled analyses showed consistent results after incorporating the hypothetical studies (as shown in Table S1). Such results revealed that the publication bias in our meta-analysis had little influence on the results.

### 3.6 Sensitivity analysis

In the analysis of ELAPE and risk of CRM, IBP and LR, sensitivity analyses using the *“metaninf” Stata* command (Figure S2a-S2c) indicated multicenter studies, such as Klein, West, and Ortiz^[11,19,33]^ were the main causes of the heterogeneity in the corresponding group. The heterogeneity vanished or was decreased after removing the studies which may be the origin of heterogeneity, while the association still kept significant except for the LR analysis (Figs. 2–4). In addition, no other study influenced the pooled RR qualitatively as indicated by the sensitivity analyses, as shown in Table S2.

## 4 Discussion

ELAPE has been advocated in recent years. As described in previously,^[14]^ the main differences between this procedure and conventional APE are as follows: the mesorectum is not dissected off of the levator muscles; the perineal portion of the operation is performed with the patient in the prone jack-knife position; and the entire levator muscle is resected en bloc with the anal canal and lower rectum, which creates a cylindrical specimen with removal of more tissue surrounding a distal rectal cancer.^[9,14]^ Early reports of the ELAPE technique had effect in reducing the occurrence of CRM involvement, IBP, and LR when compared with conventional APE.^[6,7]^ In our analysis, the efficacy differences between APE and ELAPE were conclusive. The application of ELAPE resulted in significant reductions in risk of IBP involvement, 3-year mortality, insignificant increases in risk of CRM positivity and LR. Nevertheless, an inconsistent result was got on the correlations between risk of CRM and ELAPE versus APE.

It is known that IBP and tumor involvement of the CRM are strong predictors of postoperative LR and survival in rectal cancer, LR of rectal cancer may result in severe outcomes, which are associated with severely disabled symptoms and treatment difficulty.^[48,49]^ For this reason, the ELAPE technique had the potential to substantially improve patient outcomes by reducing the incidence of CRM involvement and IBP.^[6,11]^ In our analysis, ELAPE produced a favorable outcome in reducing the risk of no more than 3 years LR, but insignificant increase in the overall LR or more than 3 years LR. Therefore, the evidence that the application of ELAPE reduces the risk of LR is sufficient to some extent. In addition, 3-year survival benefit may be attributed to the agreement that a radical resection could lead to prolonged survival with an acceptable morbidity rate.^[50,51]^

Nevertheless, the following limitations should be considered when interpreting the results of this study. First, of the 14 included studies, only one was an RCT. Therefore, the included studies cannot provide strong evidence for potential treatment effects/harm due to possible confounding factors, such as treatment (suboptimal use of pre- or postoperative treatment) or tumor characteristics (poor tumor differentiation, vascular invasion, lymphatic vessel invasion, or advanced TNM stage).^[3,13,24,51,52]^. Second, the populations of the included studies, especially the multicenter studies, were heterogeneous due to a lack of transparency in the study designs, ethnic diversity, or the lack of standardized protocols, such as the diversity of pelvic floor reconstruction methods, which may result in an overestimation or underestimation of the effects of rectal washout. Third, the surgical indications were diverse. The ELAPE procedure was not performed only in patients with T3–T4 tumors, as previously documented.^[9]^ Even T0–T2 rectal malignant neoplasms^[11,13,15,16,19]^ were treated surgically with the ELAPE technique. Moreover, in Ortiz et al,^[19]^ less than half of the patients with T0–T2 rectal cancer underwent ELAPE, which may be the cause of the heterogeneity in the corresponding analysis. Therefore, clinicians should be provided an additional incentive to consider ELAPE and should perform this surgical technique strictly based on surgical indications.

Concluding, based on these limitations, this meta-analysis supports the hypothesis that the procedure ELAPE can significantly reduce risk of 3 years LR, mortality, IBP involvement and CRM positivity when compared with conventional APE, for patients with resectable distal rectal cancer, when compared to conventional APE. Thus, the ELAPE technique is recommended to be clinically popularized and applied.

## 5 Conclusions

The application of ELAPE is more effective in reducing the chance of 3 years LR, mortality, IBP involvement and CRM positivity when compared with conventional APE, irrespective of heterogeneity among the included studies. Thus, the procedure ELAPE is worthy of being widely applied in clinic.

## 6 Author contributions

Conceived of and designed the experiments—YZ, CZ, DW; performed the experiments—YZ, CZ, XM, BS; analyzed the data—YZ, DW, BW, BS, and LZ; contributed reagents/materials/analysis tools—YY, BW, XM; wrote the paper—YZ, BW, CZ; reviewed/edited the manuscript—CZ, YY, and LZ.

## Supplementary Material

Supplemental Digital Content
