# Supplementary material for: Standard versus extralevator abdominoperineal excision and oncologic outcomes for patients with distal rectal cancer: A meta-analysis
Source: Medicine (Baltimore). 2017 Dec 29;96(52):e9150. doi: 10.1097/MD.0000000000009150 (PMC6393134; doi:10.1097/MD.0000000000009150)
Supplement: Supplemental Digital Content [file medi-96-e9150-s001.doc]

Figure S1a Funnel plots for risk of local recurrence


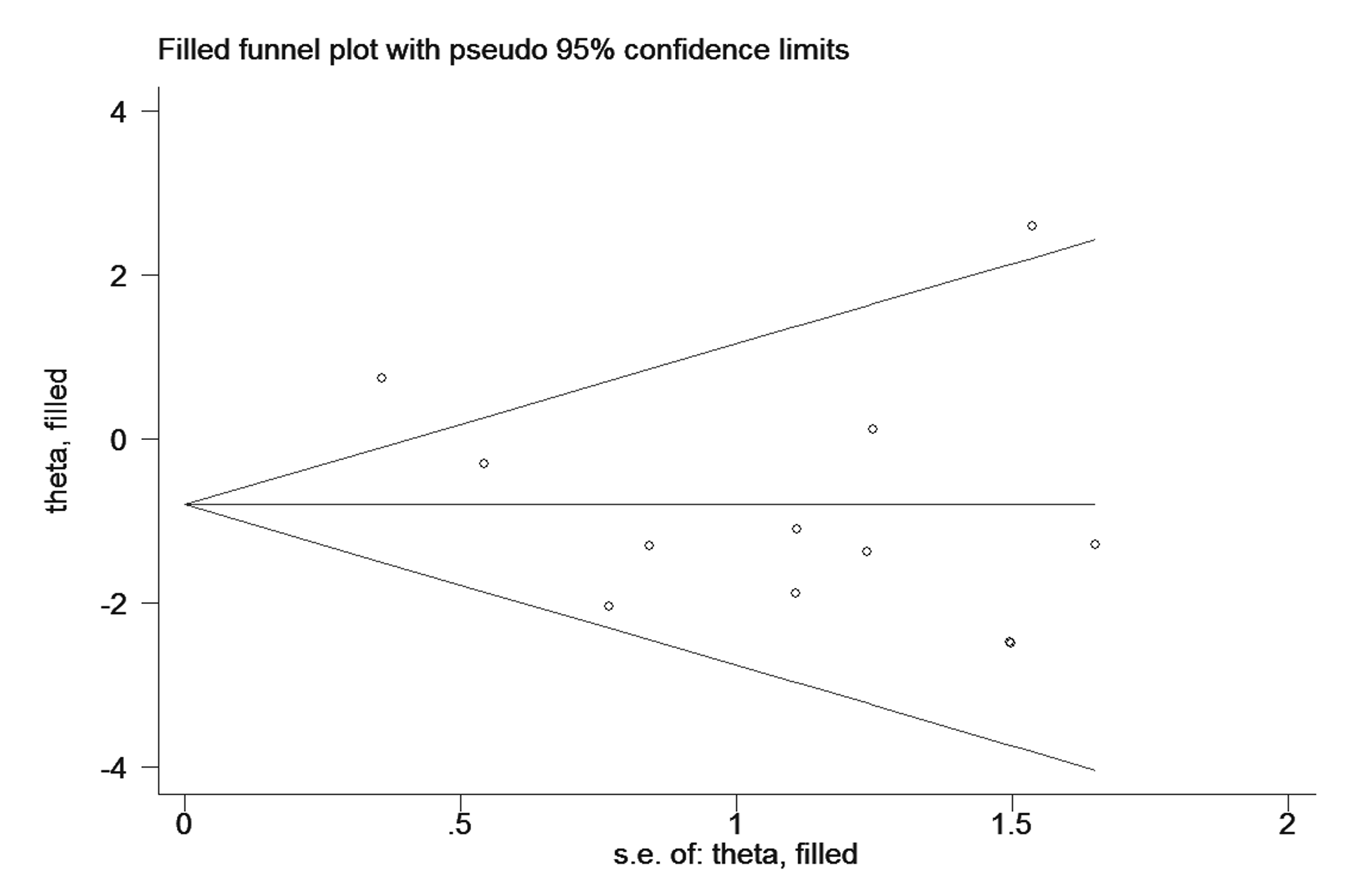


Figure S1b Funnel plots for risk of three year mortality


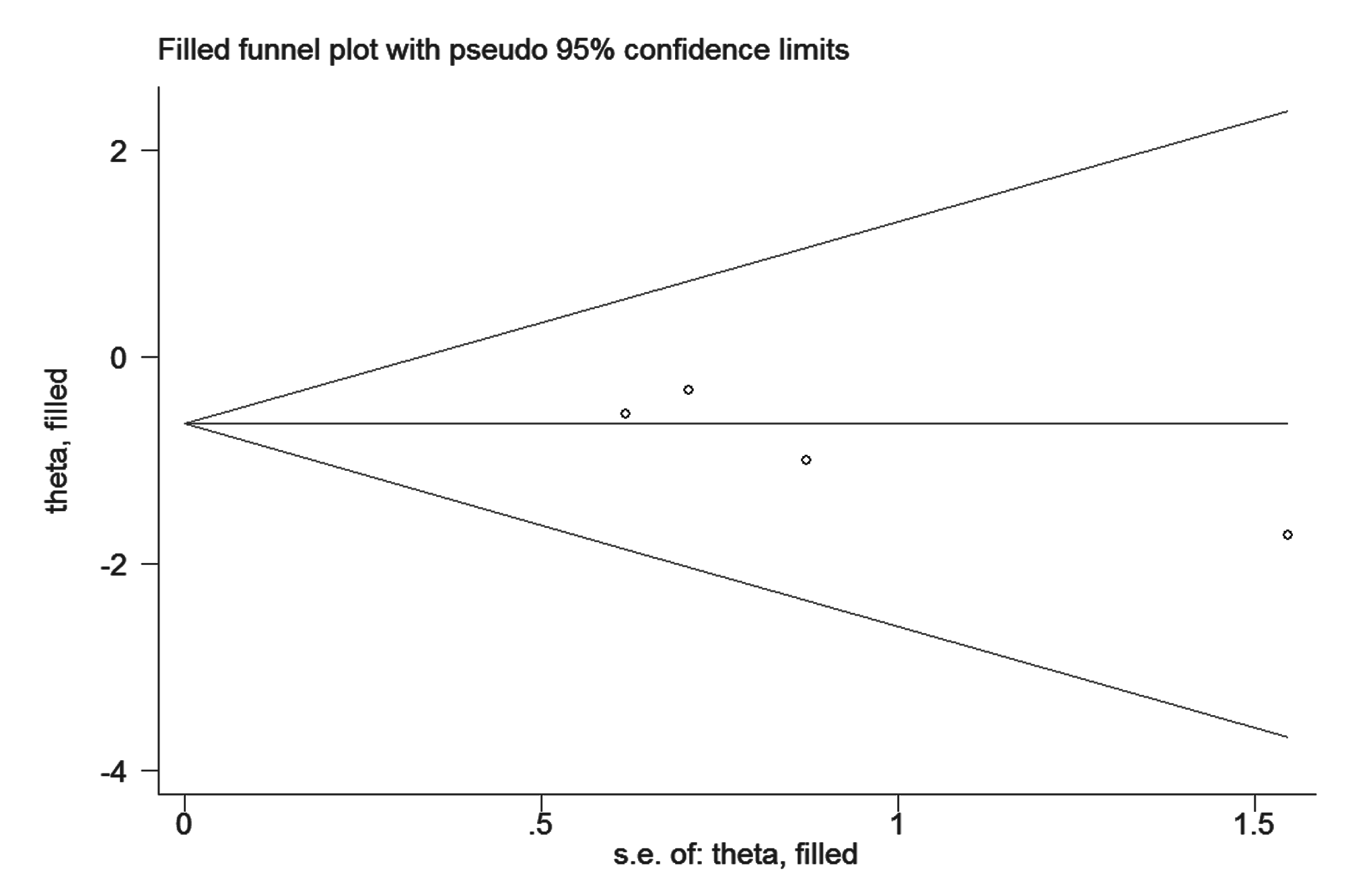


Figure S1c Funnel plots for risk of CRM


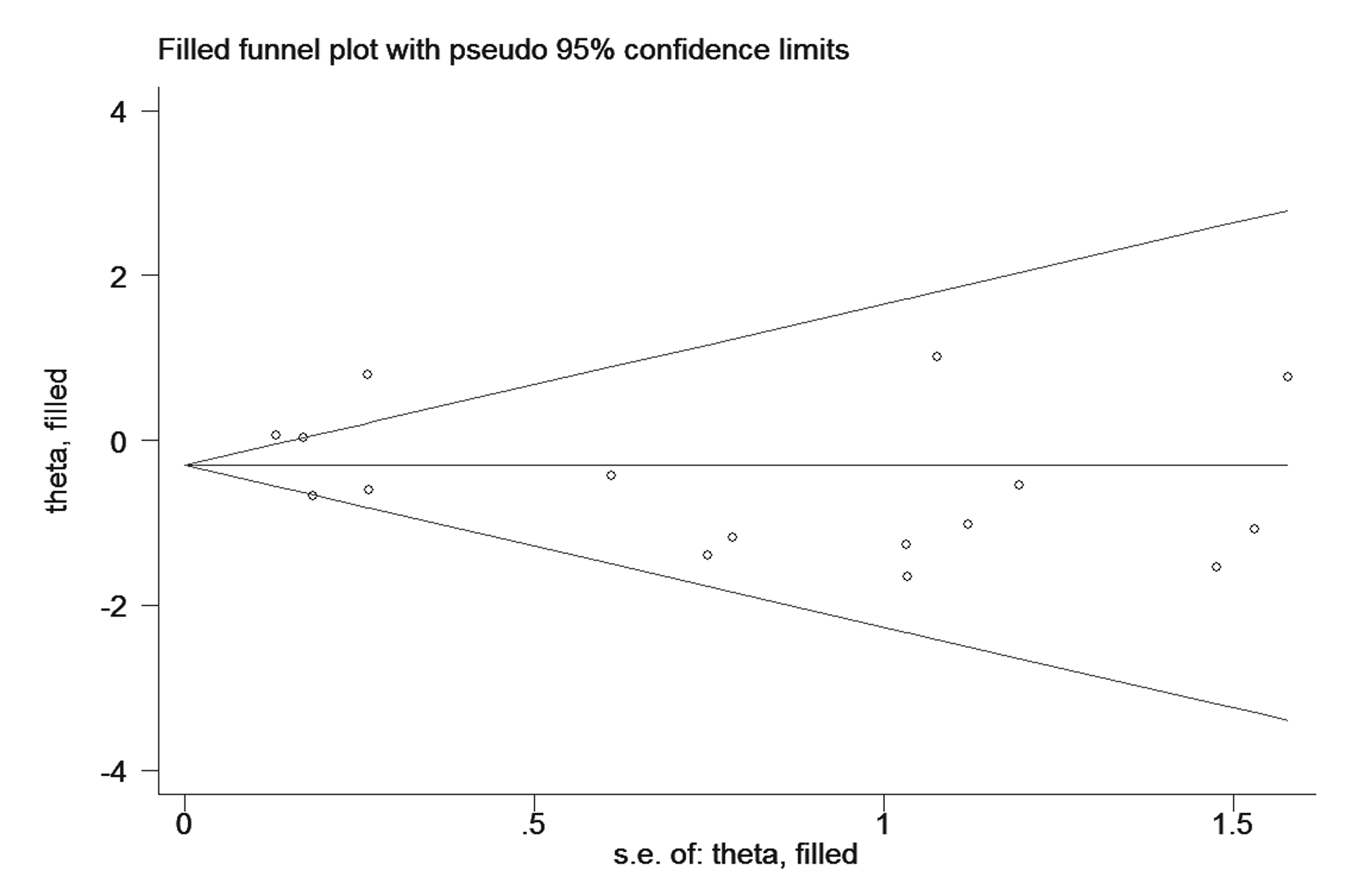


Figure S1d Funnel plots for risk of IBP


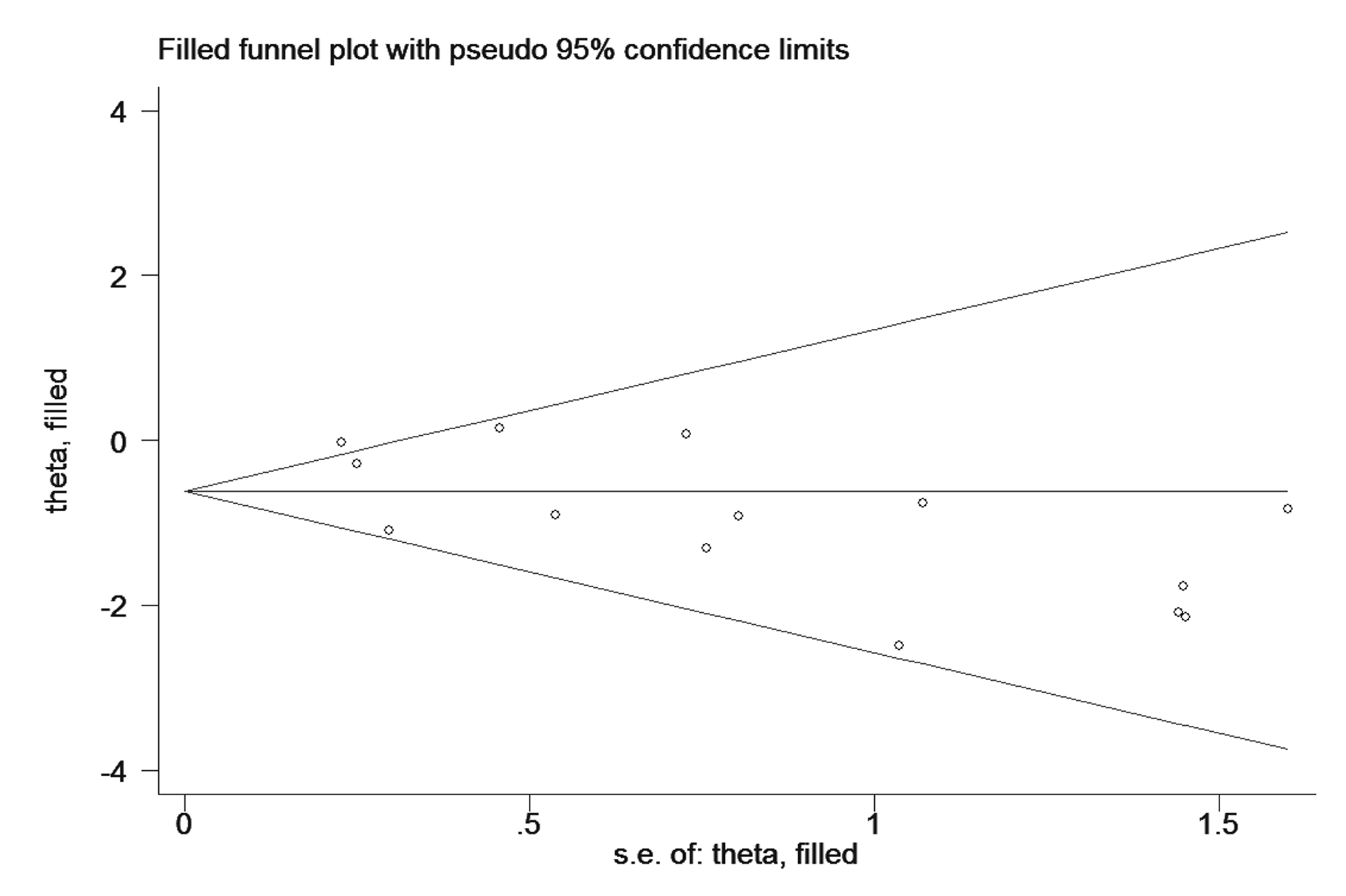


Figure S2a Sensitivity analysis for risk of local recurrence


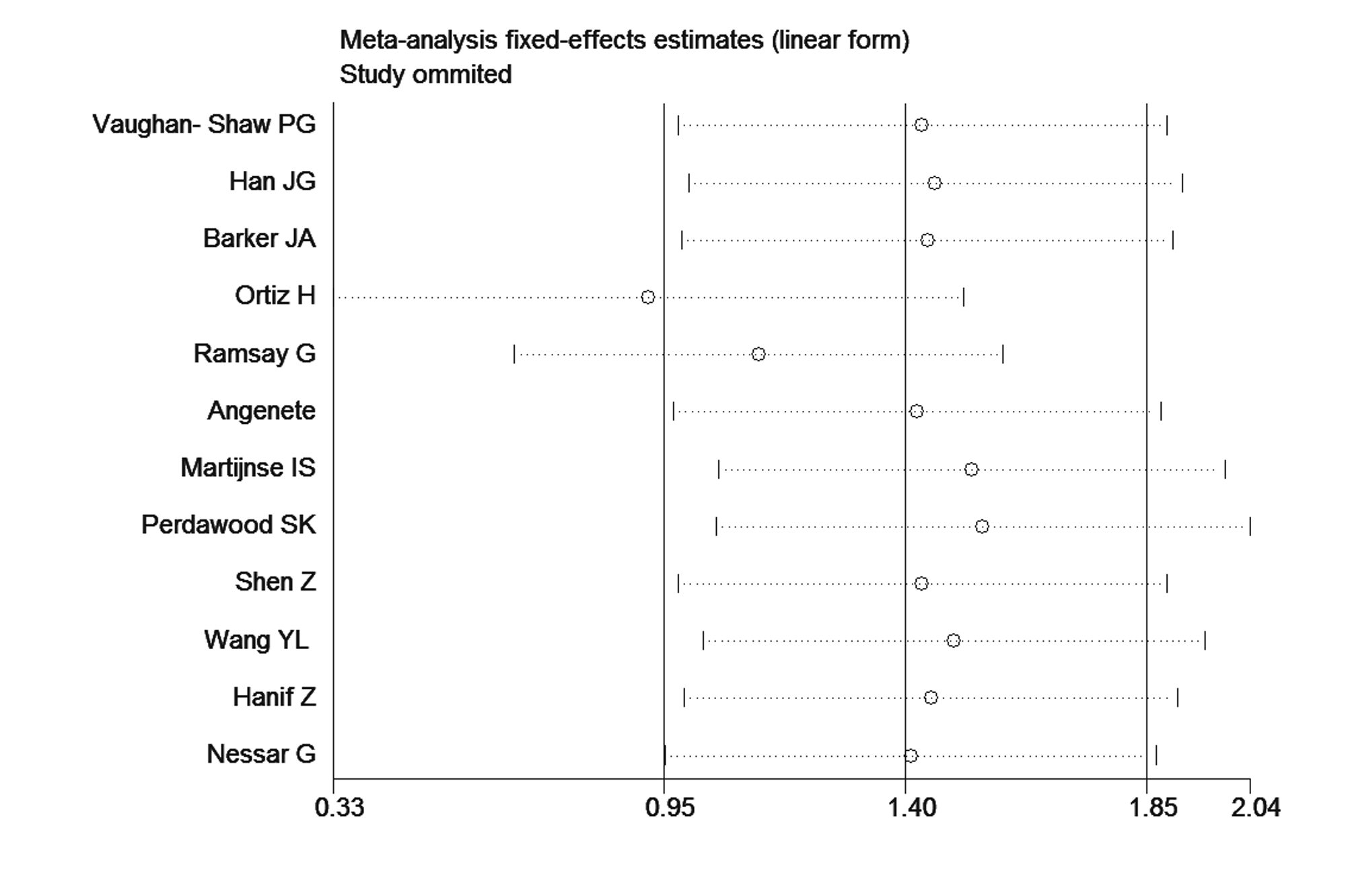


Figure S2b Sensitivity analysis for risk of CRM


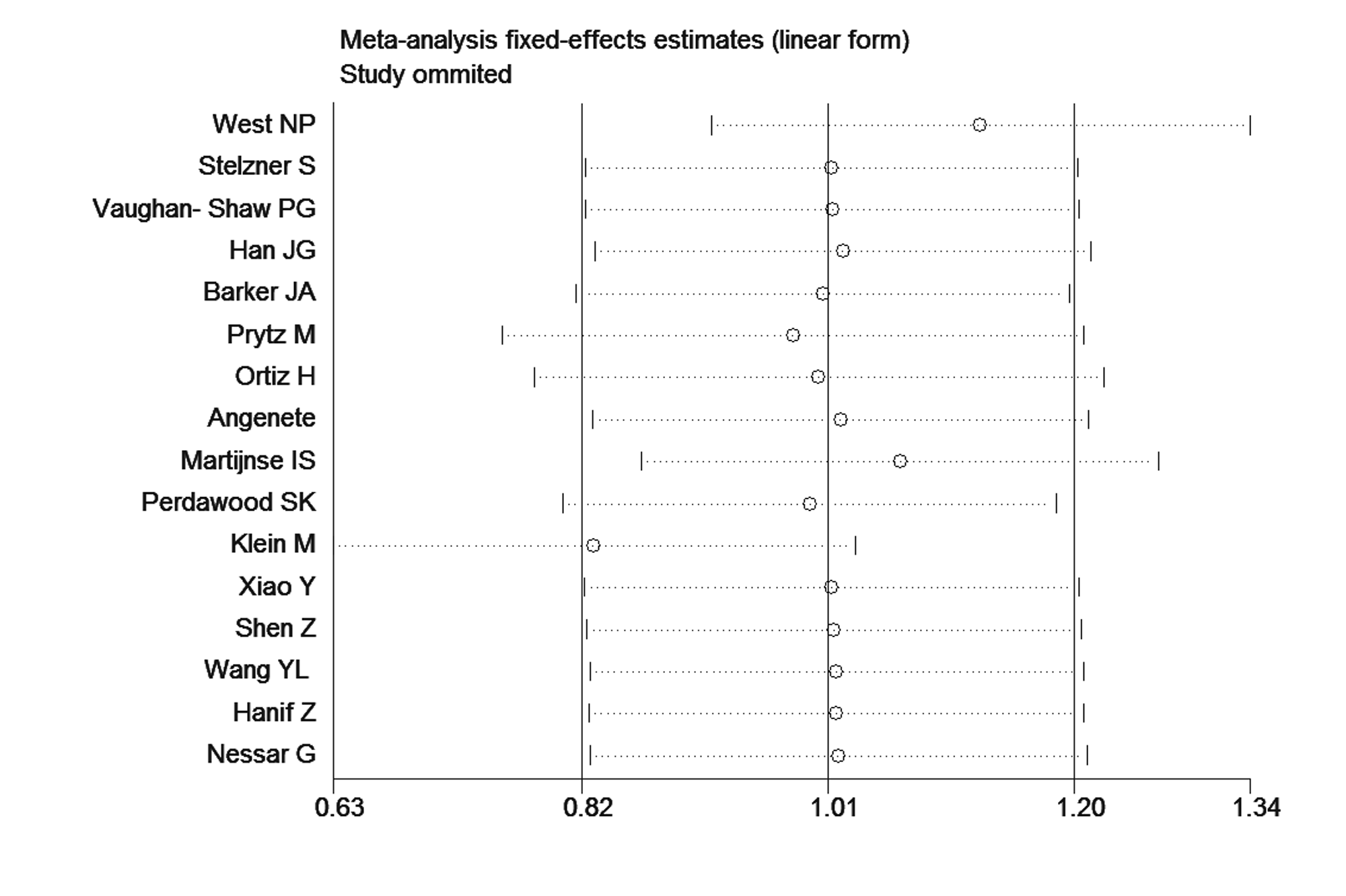


Figure S2c Sensitivity analysis for risk of IBP


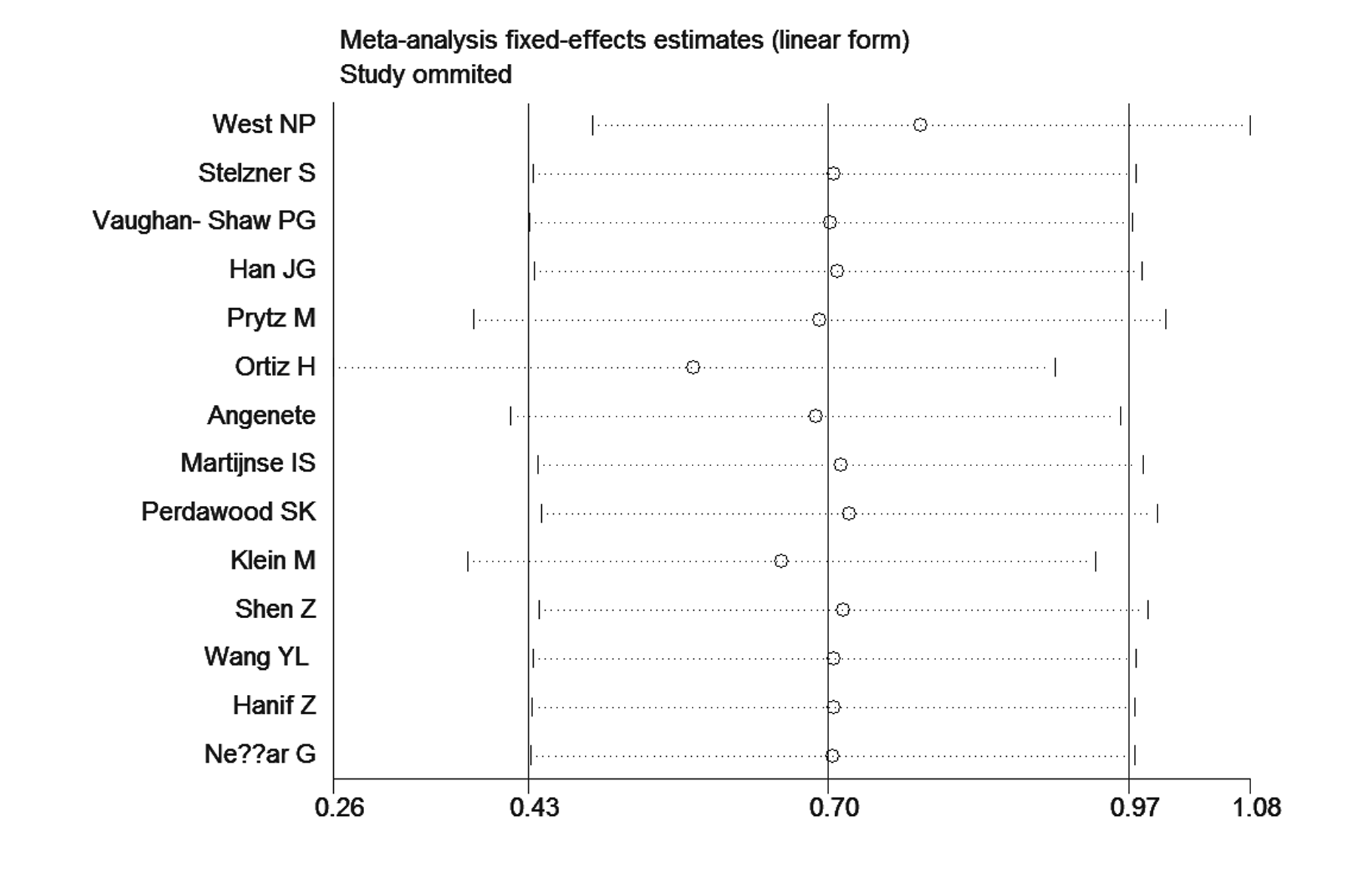


Table S1. Results of Egger's test

| Items | Outcomes of interest | | | | | | | |
| --- | --- | --- | --- | --- | --- | --- | --- | --- |
|  | LR | | Three-year mortality | | CRM | | IBP | |
|  | slope | bias | slope | bias | slope | bias | slope | bias |
| Coef. | 0.9321841 | -1.759494 | 0.4336541 | -1.429504 | 0.092502 | -0.884288 | 0.9321841 | -1.759494 |
| Std. Err. | 0.6219366 | 0.7811051 | 0.4457586 | 0.5613486 | 0.2403438 | 0.6191961 | 0.6219366 | 0.7811051 |
| t | 1.5 | -2.25 | 0.97 | -2.55 | 0.38 | -1.43 | 1.5 | -2.25 |
| P>|t| | 0.165 | 0.048 | 0.433 | 0.126 | 0.706 | 0.175 | 0.165 | 0.048 |
| 95% CI |  |  |  |  |  |  |  |  |
| LL | -0.453577 | -3.499904 | -1.48429 | -3.844792 | -0.4229843 | -2.212331 | -0.453577 | -3.499904 |
| Ul | 2.317945 | -0.0190829 | 2.351599 | 0.985784 | 0.6079882 | 0.4437557 | 2.317945 | -0.0190829 |

Table S2. Results of sensitivity

| Items | Outcomes of interest | | | | | | | |
| --- | --- | --- | --- | --- | --- | --- | --- | --- |
|  | LR | | Three-year mortality | | CRM | | IBP | |
|  | Fixed | Random | Fixed | Random | Fixed | Random | Fixed | Random |
| ORs or RRs | -0.289 | -0.805 | -0.653 | -0.653 | -0.17 | -0.394 | -0.514 | -0.704 |
| 95% CI |  |  |  |  |  |  |  |  |
| Lower | -0.739 | -1.636 | -1.431 | -1.431 | -0.36 | -0.82 | -1.028 | -1.408 |
| Upper | 0.162 | 0.026 | 0.125 | 0.125 | 0.02 | 0.031 |  |  |
| Asymptotic |  |  |  |  |  |  |  |  |
| z-value | -1.256 | -1.899 | -1.645 | -1.645 | -1.751 | -1.816 | -3.758 | -3.216 |
| p-value | 0.209 | 0.058 | 0.1 | 0.1 | 0.08 | 0.069 | 0 | 0.001 |
